# Three-dimensional visualization of cerebral blood vessels and neural changes in thick ischemic rat brain slices using tissue clearing

**DOI:** 10.1038/s41598-022-19575-w

**Published:** 2022-09-23

**Authors:** Eun-Joo Lee, Sung-Kuk Hong, Dong-Hwa Choi, Sang-Il Gum, Mee Yul Hwang, Dong Sun Kim, Ji Won Oh, Eun-Shil Lee

**Affiliations:** 1 Binaree, Inc., STE#608 Daegu Techbiz Center, Techno Gongwon-Ro 16, Dalseong-Gun, Daegu, 43017 South Korea; 2 Department of Anatomy, School of Medicine, Kyungpook National University, Gukchaebosang-Ro 680, Jung-Gu, Daegu, 41944 South Korea; 3 Biocenter, Gyeonggido Business & Science Accelerator, Gwanggyo-Ro 107, Yeongtong-Gu, Suwon, 16229 South Korea; 4 Department of Anatomy, Yonsei University College of Medicine, Yonsei-Ro 50, Seodaemun-Gu, Seoul, 03722 South Korea; 5 Graduate School of Medical Science, Brain Korea 21 Project, Yonsei University College of Medicine, Yonsei-Ro 50, Seodaemun-Gu, Seoul, 03722 South Korea

**Keywords:** Diseases of the nervous system, Stroke

## Abstract

Blood vessels are three-dimensional (3D) in structure and precisely connected. Conventional histological methods are unsuitable for their analysis because of the destruction of functionally important topological 3D vascular structures. Tissue optical clearing techniques enable extensive volume imaging and data analysis without destroying tissue. This study therefore applied a tissue clearing technique to acquire high-resolution 3D images of rat brain vasculature using light-sheet and confocal microscopies. Rats underwent middle cerebral artery occlusion for 45 min followed by 24 h reperfusion with lectin injected directly into the heart for vascular staining. For acquiring 3D images of rat brain vasculature, 3-mm-thick brain slices were reconstructed using tissue clearing and light-sheet microscopy. Subsequently, after 3D rendering, the fitting of blood vessels to a filament model was used for analysis. The results revealed a significant reduction in vessel diameter and density in the ischemic region compared to those in contralesional non-ischemic regions. Immunostaining of 0.5-mm-thick brain slices revealed considerable neuronal loss and increased astrocyte fluorescence intensity in the ipsilateral region. Thus, these methods can provide more accurate data by broadening the scope of the analyzed regions of interest for examining the 3D cerebrovascular system and neuronal changes occurring in various brain disorders.

## Introduction

Stroke is one of the leading causes of death and disability, with approximately 88% of cases caused by a blockage of blood vessels in the brain (ischemic stroke) due to a lack of oxygen, resulting in brain cell death^1^. Because the middle cerebral artery is a common site of ischemic stroke, middle cerebral artery occlusion (MCAO) model is primarily used as a standard animal model for focal brain ischemia^2,3^.

Previous studies on stroke focused on neuronal loss, nerve damage cascade, neural cell rescue, and specific neuronal reorganization caused by ischemic brain injury^4–8^. However, because decreased blood supply to the brain leads to brain damage, attempts are now being made to quantify the vascular parameters of the cerebral vascular system in disease models^9–11^. Biological and medical information obtained through the use of existing physical cutting tools, such as tomes, is limited in their ability to interpret vascular tissues and vascular system plasticity. Furthermore, data about blood vessels must be compared spatially and systematically; however, this is difficult to perform owing to extensive and dense networks in the cerebral vascular system. Magnetic resonance imaging is used to identify blood vessels and is currently advanced enough to enable quantitative analysis of microvessels^12^. However, this high-resolution approach has limitations in terms of scan time and technique.

Tissue clearing techniques, in conjunction with advanced optical imaging equipment and analytical programs, have recently been developed to identify biological information in 3D environment. An inconsistent refractive index (RI) due to the heterogeneity of chemical components within tissues results in light scattering. This phenomenon makes the tissue opaque and impossible to observe under a microscope. Several methods have been developed to reduce the RI mismatch using physical or chemical strategies, allowing the 3D structure to be explored. Recent vasculature research employs tissue clearing to interpret vascular organization and plasticity; this is accomplished by providing reliable high-resolution 3D data that is devoid of sculpted biological and medical information^13–15^. The tissue clearing technique can be divided into three major classes based on method differences: organic solvent-based, aqueous solution-based, and hydrogel-embedding methods^16,17^. First, the organic solvent-based clearing methods dehydrate and delipidate to remove water and lipids, the most abundant chemical components of biological tissue, and replace the remaining tissue with a similar RI. Dehydration reagents (such as tetrahydrofuran and tert-butanol) are typically used first, followed by lipid extraction (such as dichloromethane) and RI-matching reagents (such as benzyl alcohol/benzyl benzoate and dibenzyl ether) to make the tissue transparent. Although this method is rapid and results in tissues having high transparency, it has been reported that using an organic solvent, such as benzyl alcohol/benzyl benzoate, affects the stability of endogenous fluorescent proteins; however, new organic solvent-based methods, such as uDISCO^18^, FDISCO^19^, and sDISCO^20^, have been developed to overcome this challenge and improve the preservation of endogenous fluorescence proteins. Second, the aqueous solution-based method is classified as either a simple immersion in high-RI aqueous solutions (such as fructose, 2,2-thiodiethanol) or a method that promotes the diffusion of RI medium into tissues using hydration (such as urea) and delipidation reagents (such as triton X-100). Hydrophilic reagents, in particular, are known to preserve the 3D structure of tissue components by forming hydrogen bonds with surrounding water molecules and tissue components such as proteins. This binding conserves the fluorescent protein signal during tissue clearing^17,21^. Third, the hydrogel-embedding method is an approach to secure biomolecules through the covalent bonding of endogenous biomolecules to the hydrogel mesh. They either use harsh detergents such as SDS to remove lipids passively or electrophoresis to remove lipids. This method has been shown to reduce structural disruption and loss of native biomolecules caused by tissue-gel hybridization while preserving the fluorescent protein signal^22,23^. These tissue clearing methods have provided an optimized and efficient protocol that has evolved into a powerful 3D imaging and quantification tool.

Light-sheet fluorescence microscopy (LSFM) and confocal laser scanning microscopy (CLSM) are essential volumetric imaging tools for 3D imaging. LSFM employs an illumination system that creates a light sheet for scanning the sample and exciting fluorophores in the focus plane^24–26^. LSFM, which can produce image specimens such as the entire mouse brain, captures at a faster rate and has lesser phototoxicity and higher penetration than CLSM. However, obtaining high-resolution images of larger specimens, such as a whole rat brain, yields terabytes of image data. Alternatively, CLSM uses a pinhole system to flood the entire sample with light, eliminating out-of-focus light, thereby increasing its optical resolution^24,25^. However, exposing the entire specimen to the excitation light results in unwanted photobleaching and phototoxic effects. Taking these 3D imaging tool characteristics and image size into consideration, this study used LSFM to acquire high-resolution images of relatively large tissues from a large 3-mm-thick brain slice. CLSM was also used in tissues that required immunostaining for morphological and quantitative analysis of neuronal changes.

Rat is predominantly used as an animal model instroke research owing to its ease of surgery and similarity to human cerebrovascular anatomy and physiology. However, making the tissue transparent and capturing an image of the entire brain tissue of the rat to obtain 3D imaging and quantification data is difficult. The rat brain is larger than the mouse brain, with slightly different lipid and myelin content. Thus, 3D imaging and quantification have been developed primarily in mouse tissues rather than rat tissues. Though there are a few reports of 3D imaging of tissues subjected to endogenous fluorescence and immunostaining of rat brain using tissue clearing, there are no reports of 3D quantitative analysis of tissue vasculature. Furthermore, tissue thickness is limited by the physical and optical issues mentioned above. This study focused on the cortex and striatum of the rat brain, which are the main parts affected by the infarction. It also aimed to use aqueous-based tissue clearing to visualize and analyze the vascular and neural changes induced by MCAO in a rat model. The findings of this study will add to the existing information regarding the 3D cerebrovascular system and neuronal changes that occur in various brain disorders as well as allow an unbiased analysis of biological and clinical tissues.

## Results

### Infarction identification with 2,3,5-triphenyltetrazolium chloride (TTC) and tissue transparency using aqueous tissue clearing

**Figure 1:**
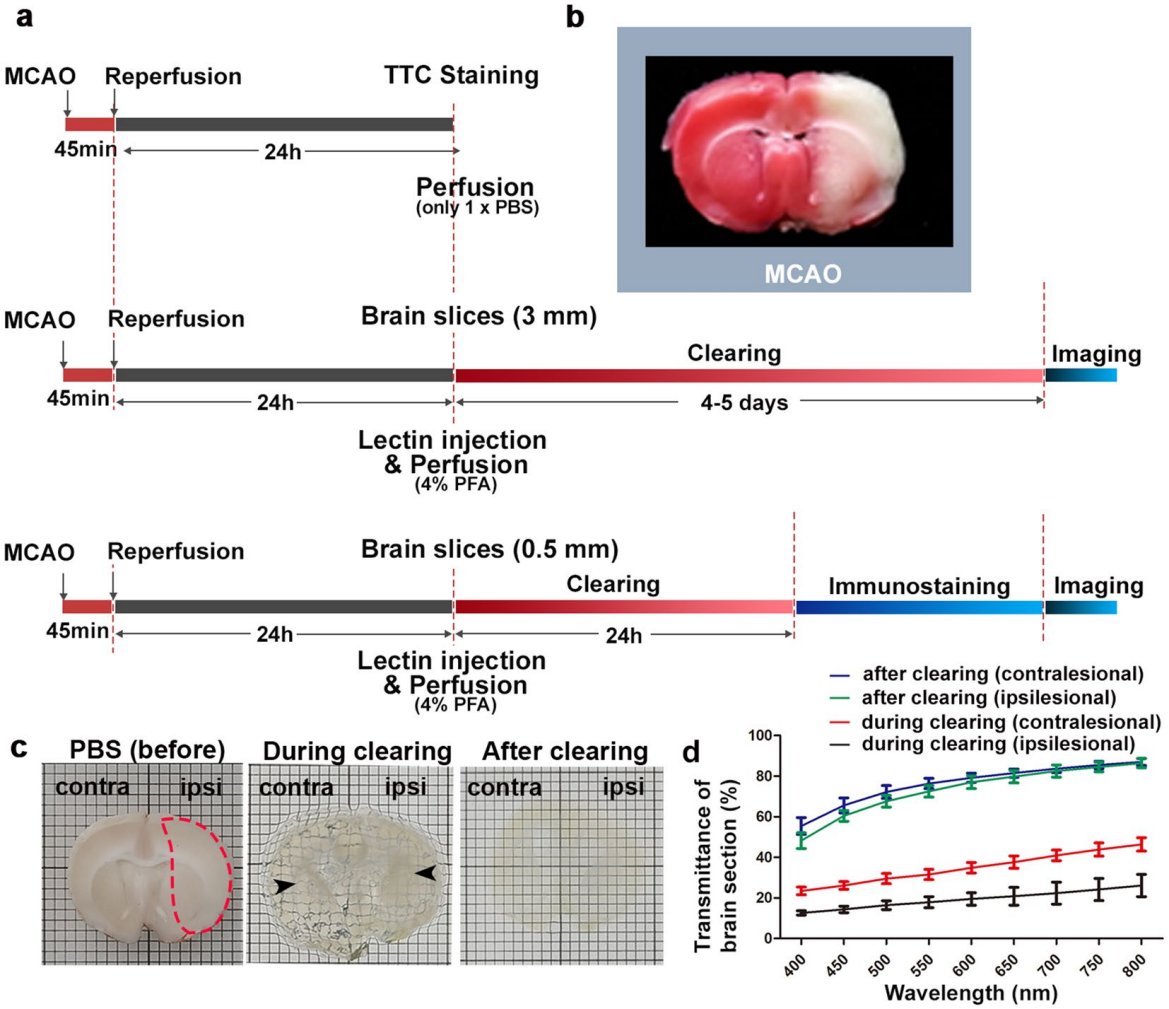
Workflow of the protocol. (**a**) Diagrams showing the overall experimental procedure. (**b**) Brain slice from MCAO rat stained with 2% TTC solution (n = 1 animal). (**c**) Bright-field images of MCAO-induced rat brain sections (3-mm-thick slice) before clearing (in PBS), during clearing (clearing solution for 24 h), and after clearing (matching RI). Grid size, 1 mm × 1 mm. The arrowheads show that the visibility of the background grid differs. (**d**) Transmittance curves of contralateral and ipsilateral brain sections during (clearing solution for 24 h) and after clearing (mean ± SD, n = 3 brain sections for each group). contra, contralesional region; ipsi, ipsilesional region.

To visualize and quantify the vascular and neural changes caused by MCAO, we stained functional vessels with tomato lectin and immunostaining with neural markers. Figure 1a depicts the entire experimental procedure. Twenty-four hours after MCAO surgery, 2-mm-thick rat brain slices were stained with TTC without paraformaldehyde (PFA) fixation, revealing hemispherical swelling and infarction in the cortex and striatum (Fig. 1b). Furthermore, the TTC staining after MCAO indicates that the rats experienced focal ischemia, implying a significant cerebral tissue injury. We used 3D visualization and analysis of blood vessels in rat MCAO models to perform aqueous-based tissue clearing methods after injecting fluorescent tomato lectin into the rat heart. Figure 1c also shows the images before (in PBS), during (clearing solution for 24 h), and after (matching RI) clearing in sequence. It can be seen in the bright-field image that the transparency of the infarct site was lower than in the contralateral area during the clearing (clearing solution for 24 h). Furthermore, the transmittance of the ipsilateral and contralateral sections was measured with a commercial spectrophotometer (Lambda 950, PerkinElmer) to quantitatively demonstrate the difference in the degree of transparency (Fig. 1d). After completing the clearing, both sides were cleared to a similar level. However, it was demonstrated that the degree of clearing differed between the ipsilateral and contralateral regions during clearing.

### 3D visualization and quantification of the blood vessels in pre-sliced rat MCAO tissues of 3-mm thickness

**Figure 2:**
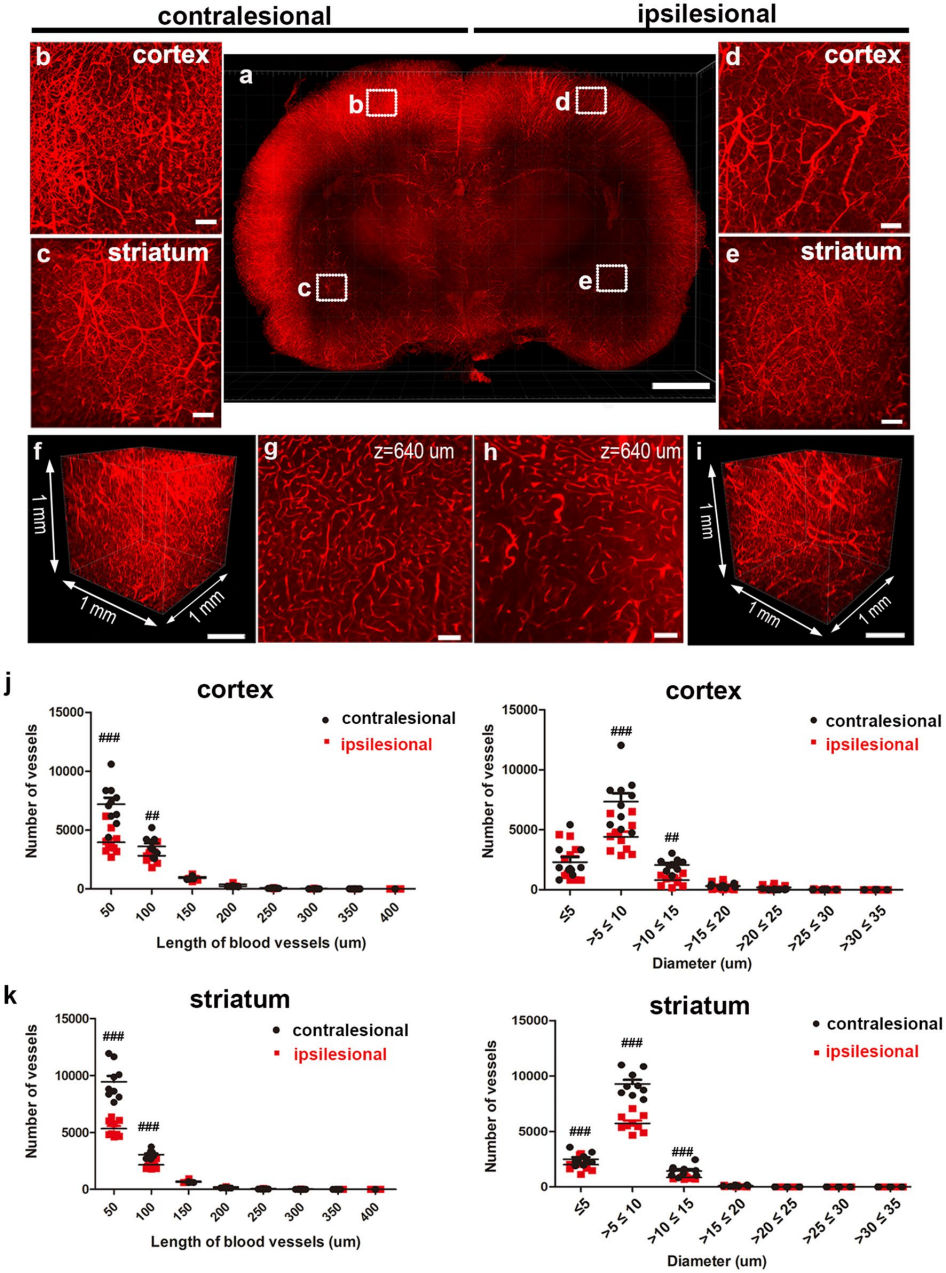
3D visualization of the vasculature in rat brain of MCAO model after tissue clearing. (**a**) 3D reconstruction of the vasculature in the 3-mm-thick rat brain slice stained with lectin. Images were obtained from LSFM equipped with a 5× objective. (**b**–**e**) Higher-magnification view of XY plane of the boxed region (cortex and striatum, imaging volume were 1 mm × 1 mm × 1 mm) in (**a**) are shown. (**b**,**c**) Maximum projection (z-stack) images of the contralesional cortex and striatum. (**d**,**e**) Maximum projection (z-stack) images of the ipsilesional cortex and striatum. (**f**,**i**) 3D reconstructions of the cortical region in (**b**,**d**) are shown. (**g**,**h**) Images at random depths of the brain slice from (**f**,**i**) are shown (Z = 640 μm relative to the top imaging surface). (**j**,**k**) Quantification of length and vessel diameter in 3–4 cortical and striatal fields of view per slice (n = 3 animals). Plots in (**j**) and (**k**) show means ± SEM values. Two-way ANOVA, FDR multiple comparison test, ^###^p < 0.001, ^##^p < 0.05. Scale bar = 2 mm in (**a**), 100 μm in (**b**–**e**,**g**,**h**), 300 μm in (**f**,**i**).

In lesion models, 3D visualization and quantitative analysis of the blood vessels in continuous and extended tissues are important indicators. Fluorescent tomato lectin perfusion was used in this study to label the cerebral vasculature 24 h after inducing stroke, followed by tissue clearing and LSFM imaging. Figure 2 and Supplementary Video 1 show 3D-rendered vasculature from 3-mm-thick coronal slices of the MCAO rat brain. Initially, the MCAO model’s ipsilesional region showed a significant decrease in blood vessel density in the cortex and striatum when compared to the contralesional region. The 3D vascular structures were also visualized in 3D images of each selected region of interest (ROI; 1 mm × 1 mm × 1 mm; shown as squares in Fig. 2a) in the contralesional and ipsilesional regions, which were fitted to a filament model using the filament tracer module of Imaris (Supplement Fig. 1). In the ipsilesional region of the MCAO model, the density of blood vessels decreased 24 h after stroke (Fig. 2b–i).

We quantified the vascular parameters in the ischemic rat brains, including the length and diameter (Fig. 2j,k). Symmetric view fields in the contralateral cortex and striatum were used as internal controls. We calculated vascular metrics by selecting 3–4 ipsilateral cortical and striatal fields (1 mm × 1 mm × 1 mm) per slice from the 3 MCAO animals. Because all of the ipsilateral parts had cerebral edema, we computed the hemispherical volume and corrected the fields in the ipsilateral cortical and striatal parts. After correction, the findings revealed that the number of blood vessels in the ipsilesional cortical and striatal regions decreased following MCAO compared to the contralateral regions. The ipsilesional region also showed a significant decrease in the number of ≤ 100-μm-long vessels and no difference in the number of ≥ 100-μm-long vessels compared with the contralesional region. Additionally, in both the cortical and striatal regions, the number of ≥ 5- and < 15-μm-diameter vessels decreased significantly in the ipsilesional region, but there was no difference in that of > 15-μm-diameter vessels. This finding revealed that microvessels with a diameter of < 15 μm and a length of < 100 μm within ipsilesional cortical and striatal regions are more affected than large blood vessels by severe MCAO.

### 3D visualization of a neural marker in pre-sliced 0.5-mm-thick MCAO rat tissues

**Figure 3:**
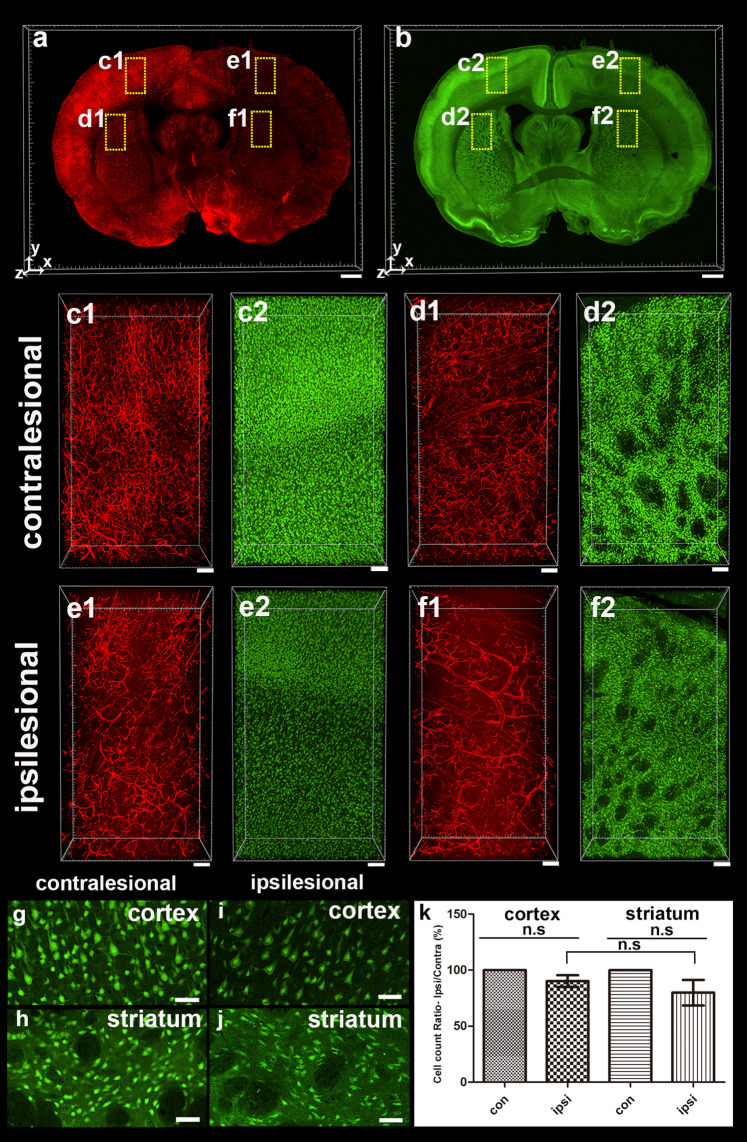
3D visualization of NeuN-positive cell loss 24 h after MCAO. (**a**,**b**) 3D reconstruction of the 0.5-mm-thick rat brain slice stained with lectin (**a**) and NeuN (**b**). (**c**–**f**) Higher-magnification views of the boxed regions in (**a** and **b**) are shown. Vascular density loss and NeuN-positive cell loss were noted in the ipsilesional cortex and striatum compared with the contralateral side. (**g**–**j**) Morphological change of NeuN-positive cells in ipsilesional cortex and striatum compared with the contralateral side. (**k**) Graph of the ratio of NeuN-positive cells obtained using automated cell count in the ipsilateral areas to the corresponding contralateral areas of the cortex and striatum 24 h after ischemia using a spot detection strategy generated with Imaris v.9.5.0 software (measures 3–4 cortical and striatal fields per animal, n = 3 animals). Data are presented as the mean percentage relative to corresponding contralateral areas ± SEM. No significant differences were observed in the ratio of NeuN-positive cells between the ipsilateral cortex and the striatum. Paired t-test, FDR multiple comparison test, n.s., no significant difference. Scale bar = 1 mm in (**a**,**b**), 100 μm in (**c**–**f**) and 50 μm in (**g**–**j**).

For evaluating MCAO-induced neural damage, we performed immunostaining using various neuronal markers (NeuN, parvalbumin [PV], and microtubule-associated protein 2 [MAP2]) and a glial marker (glial fibrillary acidic protein [GFAP]) on the 0.5-mm-thick brain slices of rat MCAO models 24 h after ischemia induction. Considerable changes in the expression patterns of these markers were observed at the core of the ischemic region compared with the contralesional non-ischemic regions. Fluorescence intensities of lectin and NeuN-positive cells were generally weaker in NeuN-stained tissue than in the contralateral region by MCAO-induced infarction (Fig. 3a,b). Furthermore, the reduction in blood vessel density in the cortex and striatum resulted in morphologically different densities in NeuN-positive cells (Fig. 3c–j). These cells had regular shapes, such as a round or oval morphology, and occasionally appeared as axons in contralesional regions. In contrast, NeuN-positive cells in the ipsilateral cortex had distorted morphologies and more irregular shapes (rather than a typical round or oval shape) and fewer axons. Before performing the quantitative analysis of NeuN-positive cells in both regions, two-dimensional (2D) manual counting and 3D analysis of NeuN-positive cells were performed at specific ROIs (Supplement Fig. 2). This is an important process for confirming the difference in counting methodology between them by comparing the existing 2D analysis before the 3D analysis. We found that the number of NeuN-positive cells in the cortex 24 h after MCAO decreased slightly at similar levels in both cell counting methods.

Our findings revealed a nearly 20% decrease in ipsilateral cortical and striatal NeuN-positive cells when compared with contralateral cerebral and striatal regions, indicating a region-specific difference. There was no statistically significant difference between the cortex and striatum during the loss of NeuN-positive cells caused by severe MCAO (Fig. 3k). The total vessel length decreased compared to that of the contralateral region in all selected ROIs (71%–74% of the contralateral region), without a region-related difference, owing to a similar decrease in the cortex and striatum.

**Figure 4:**
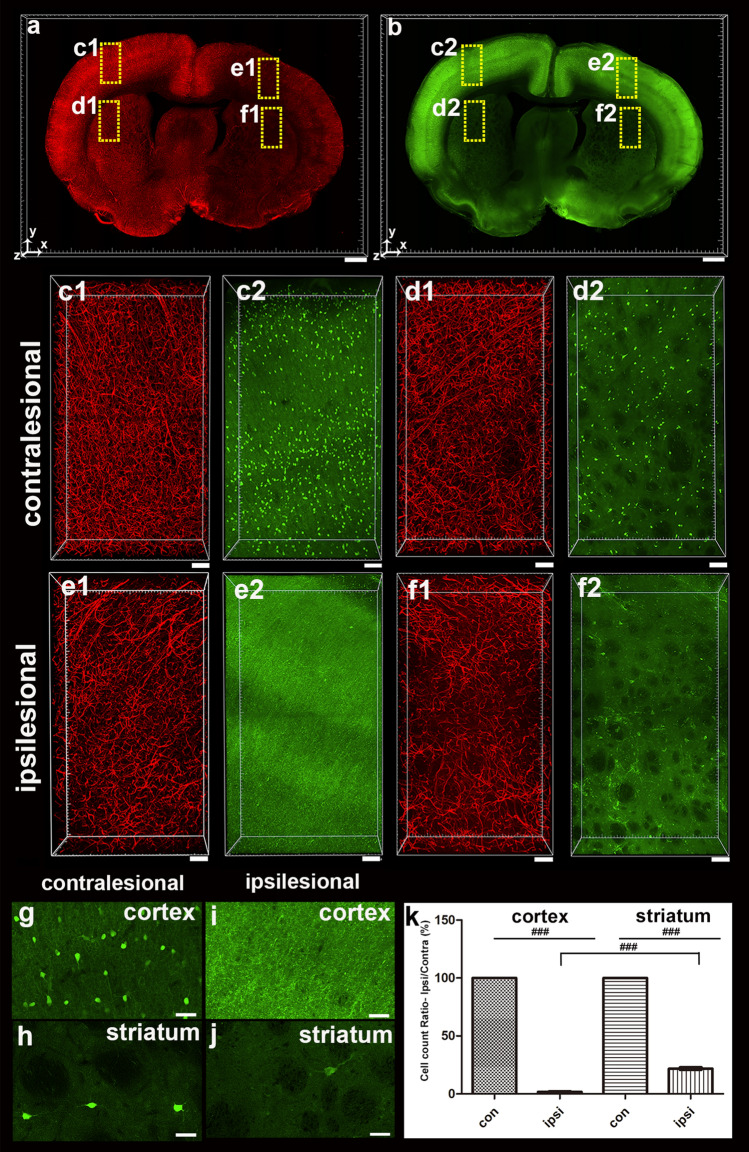
3D visualization and quantification of PV-positive cell and vascular reduction 24 h after MCAO. (**a** and **b**) 3D reconstruction of the 0.5-mm-thick rat brain slice stained with lectin (**a**) and PV (**b**). (**c**–**f**) Higher-magnification views of the boxed regions in (**a**,**b**) are shown. There was a vascular density loss and PV-positive cell loss in the ipsilesional cortex and striatum compared with the contralateral side. (**g**–**j**) Morphological change in PV-positive cells in the injured cortex and striatum compared with the contralateral side. (**k**) Graph of the ratio of PV-positive cells using automated cell count in the ipsilateral areas to the corresponding contralateral areas of the cortex and striatum 24 h after ischemia using a spot detection strategy generated with Imaris v.9.5.0 software (measures 3–4 cortical and striatal fields per animal, n = 3 animals). Data are presented as the mean percentage relative to corresponding contralateral areas ± SEM. Paired t-test, FDR multiple comparison test, ^###^*p* < 0.001, Scale bar = 1 mm in (**a**,**b**), 100 μm in (**c**–**f**), 50 μm in (**g**–**j**).

Because PV-positive neurons are vulnerable in the disease model, immunostaining with PV antibody was performed in comparison to the contralateral area. The fluorescence intensity of lectin was decreased as expected in blood vessel staining and immunostaining using lectin and PV antibody, respectively, compared to the contralateral area by MCAO infarction, although PV-positive fluorescence intensity was slightly increased in the cortical region (Fig. 4a,b). The MCAO procedure also reduced the number of vessels at 24 h compared to the contralateral cerebral and striatal regions, reducing the number of PV-positive neurons (Fig. 4c–f). Morphologically accurate cell bodies and processes were observed in the contralateral cortex, and neurites were observed despite a weak fluorescence signal (Fig. 4g). In the ipsilateral cortex, finding cell bodies was difficult; nevertheless, neurites and axons were prominent (Fig. 4i). Both regions had similar fluorescence intensity in the striatum; however, the number of PV-positive cells significantly decreased in the ipsilateral region, and the cell shape was also altered (Fig. 4h,j). The data from two cortical and striatal regions showed that the number of PV-positive cells in the cortex 24 h after MCAO markedly decreased to comparable levels in the two cell counting methods (Supplement Fig. 2). We found that the vessel length in the cortex and striatal regions was only about 65%–70% of that in the contralateral region. Compared with the contralateral cerebral and striatal regions, our data showed a > 98% decrease in the number of PV-positive cells in the cortex and a nearly 80% decrease in striatal cells, indicating a region-specific difference (Fig. 4k).

**Figure 5:**
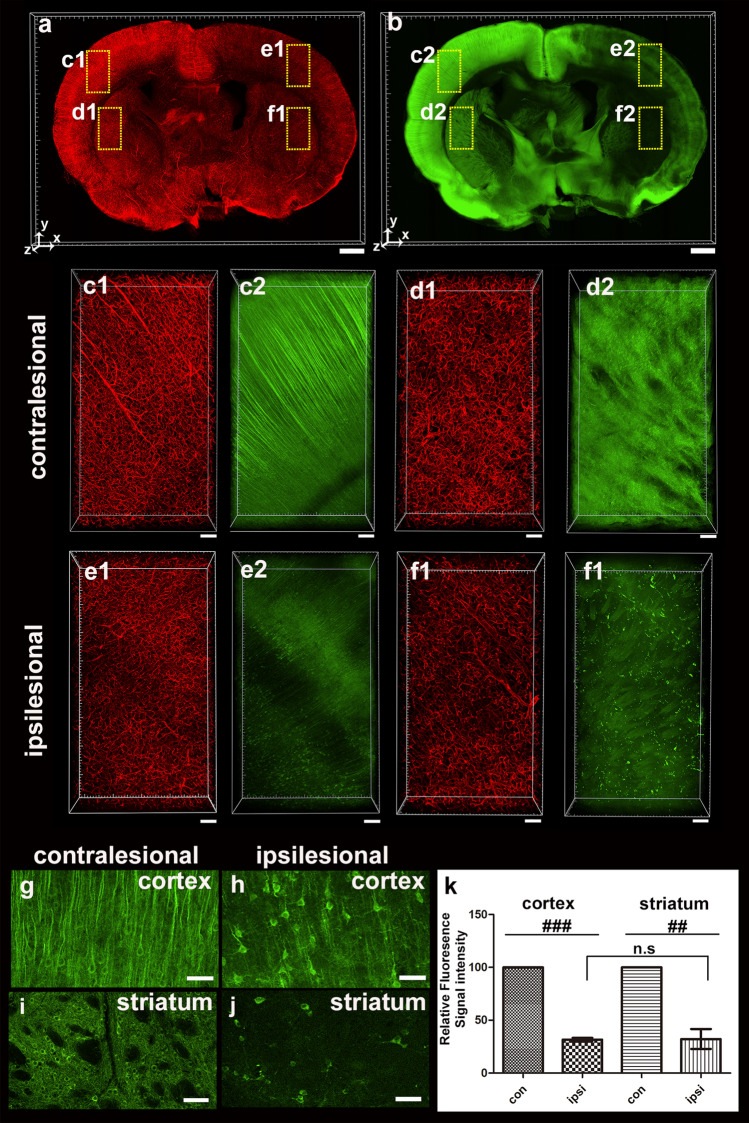
3D visualization and quantification of MAP2-related immunofluorescence intensities 24 h after MCAO. (**a**,**b**) 3D reconstruction of the 0.5-mm-thick rat brain slice stained with lectin (**a**) and MAP2 (**b**). (**c**–**f**) Higher-magnification views of the boxed regions in (**a**,**b**) are shown. Vascular density loss and reduction in MAP2-positive fluorescence intensity were observed in the injured cortex and striatum compared with the contralateral side. (**g**–**j**) Morphological change of MAP2-positive cells in the injured cortex and striatum compared with the contralateral side. (**k**) Graph of the ratio of MAP2-related fluorescence intensity of the ipsilateral versus contralateral cortex 24 h after MCAO injury. MAP2-related fluorescence intensities in the infarction area were significantly decreased. Data are presented as the mean percentage relative to corresponding contralateral areas ± SEM (measures 3–4 cortical and striatal fields per animal, n = 3 animals). A significant difference was observed in fluorescence intensity of MAP2-positive cells between the contralesional and ipsilesional cortex, or striatum. No significant differences were observed in the fluorescence intensity of MAP2-positive cells between the ipsilateral cortex and the striatum. Paired t-test, FDR multiple comparison test, ^###^*p* < 0.001, ^##^*p* < 0.05. n.s = no significant differences. Scale bar = 1 mm in (**a**,**b**), 100 μm in (**c**–**f**), 50 μm in (**g**–**j**).

Compared with contralateral control regions, the intensity of MAP2-related immunofluorescence decreased 24 h after ischemia induction. The boundary of the ischemic lesion is indicated by a clear-cut loss of MAP2-positive immunolabeling (Fig. 5a,b). The MCAO procedure also reduced fluorescence intensity and the number of blood vessels at 24 h when compared to contralateral cortical and striatal regions, particularly the significant reduction of MAP2-positive neurons (Fig. 5c–f). After ischemia, MAP2-positive cells exhibited morphological changes in neurites. Our results showed that MAP2 immunoreactivity was intact in the contralateral cortical region of MCAO; however, the number of MAP2-positive cell bodies and apical dendrites in the ipsilateral cortex was significantly reduced at 24 h. (Fig. 5g,h). MCAO dimmed many round MAP2-positive cell bodies in the striatum (Fig. 5i,j). Because the MAP family member is strongly expressed primarily in neuronal dendrites, we measured the immunofluorescence intensity of the MAP2-positive rather than counting neural cells to quantify the reduction in MAP2 (Fig. 5k). The MAP2-positive fluorescence intensity was approximately 31.68% ± 3.72% and 32.20% ± 21.11% of the control in the ischemic-induced cortex and ischemic-induced striatal region, respectively, and the total length of the blood vessels was approximately 72% (72.86% ± 4.58%) and 75% (75.35% ± 2.43%) of the control in the ischemic-induced cortex and ischemic-induced striatal region, respectively. There was no significant difference between the ipsilateral cortex and ipsilateral striatum in the fluorescence intensity of MAP2-positive cells induced by severe MCAO.

**Figure 6:**
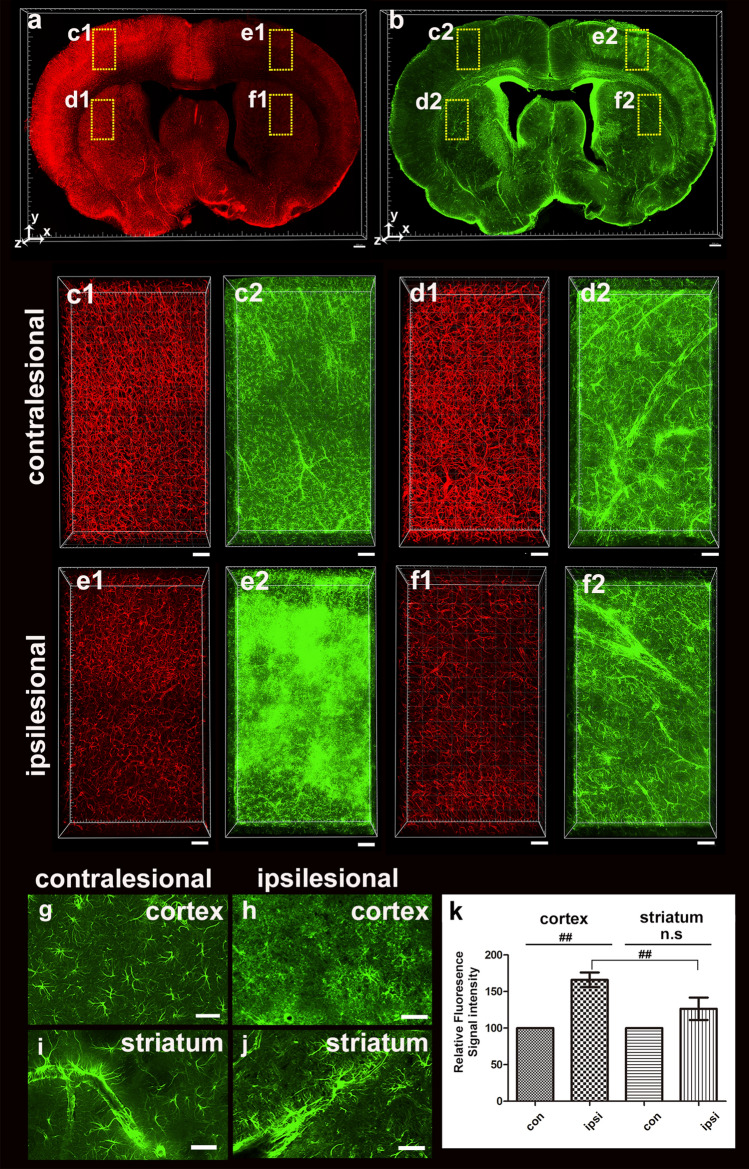
3D visualization and quantification of GFAP-related immunofluorescence intensities 24 h after MCAO. (**a**,**b**) 3D reconstruction of the 0.5-mm-thick rat brain slice stained with lectin (**a**) and GFAP (**b**). (**c**–**f**) Higher-magnification views of the boxed regions in (**a**,**b**) are shown. A vascular density loss and increased GFAP-positive fluorescence intensity were observed in the ipsilesional cortex compared with the contralateral side. (**g**–**j**) Morphological change of GFAP-positive cells in the ipsilesional cortex and striatum compared with the contralateral side. (**k**) Graph of the ratio of GFAP-related fluorescence intensity of the ipsilateral versus contralateral cortex 24 h after MCAO injury. GFAP-related fluorescence intensities in the cortex are significantly increased. Data are presented as the mean percentage relative to corresponding contralateral areas ± SEM (measures 3–4 cortical and striatal fields per animal, n = 3 animals). Significant differences were observed in fluorescence intensity of GFAP-positive cells between the contralesional and ipsilesional cortex, but no significant differences were observed between the contralesional and ipsilesional striatums. In addition, significant differences were observed in the fluorescence intensity of GFAP-positive cells between the ipsilateral cortex and the striatum. Paired t-test, FDR multiple comparison test, ^##^*p* < 0.05. n.s = no significant differences. Scale bar = 1 mm in (**a** and **b**), 100 μm in (**c**–**f**), 50 μm in (**g**–**j**).

We also performed immunostaining with the GFAP astrocyte marker. The fluorescence intensity of blood vessels stained with lectin decreased compared with the contralateral region, while the fluorescence intensity of GFAP increased significantly in the ischemic cortical areas rather than the ischemic striatum (Fig. 6a–f). The boundary of the ischemic region was identified because of a sharp increase in immune markers associated with GFAP. We found that glial scars in a large cortex 24 h after ischemia in the severe acute ischemic brain. GFAP-relative signals were found in the soma and the main processes of astrocytes in the contralateral region. However, although GFAP-positive cells were observed in the ipsilateral cortex, glia scarring cannot always identify the morphological characteristics of GFAP-positive cells. Compared with the contralateral region, the GFAP-positive cells exhibited hypertrophy in the ipsilateral striatal region and decreased branching pattern complexity (Fig. 6g–j). In this study, there was a significant increase in fluorescence intensity in the ischemic cortex (166% ± 22.66%) compared with the contralateral region but not in the striatum (126% ± 34.26%) (Fig. 6k). The fluorescence intensity of GFAP-positive cells by severe MCAO differed significantly between the ipsilateral cortex and the ipsilateral striatum. The total blood vessel length was also found to be approximately 73% (73.59% ± 4.43%) in the cortex and 69% (69.06% ± 6.28%) in the striatum when compared to the contralateral region.

## Discussion

In this study, we examined the vascular and neural changes caused by MCAO in large volume tissues of a rat model. Unlike previous studies, a tissue clearing method was used to minimize the loss of structural information through the processing of thin specimens. Using this method, blood vessels were visualized in 3D under a high-resolution microscope and quantified with an analytical program. Because immunostaining is compatible with tissue clearing, neural markers and blood vessels were quantified simultaneously.

Previous studies demonstrated the use of vascular staining dyes and markers as a compatible method with tissue clearing^15,27–31^. Fluorescent lectin is a vascular staining marker used in lesion studies and primarily for stroke-induced remodeling^32,33^. Lectin-based vascular staining involves injecting the lectin directly into the tail vein, cannula implanted in the jugular vein, directly into the left ventricle, or lectin staining outside of the tissue. The stained endothelial cells in this study were only in perfused vessels by transcardial administration of tomato lectin to identify only the functional vessels. In the ipsilateral region compared with the corresponding contralateral region of the MCAO model, it was demonstrated that the fluorescence intensity of perfused lectin significantly decreased. Therefore, presumably, the occluded microvessels caused by stroke induction interfered with the flow of lectin, or leakage to the brain parenchyma occurred due to the loosening of the blood–brain barrier integrity in the microvessels.

Because vascular analysis is important in functional and pathological studies, 3D analysis of an intact vascular system with tissue clearing reduces topological data loss and increases accuracy. Therefore, many researchers employ 3D visualization and analysis of the vasculature. Normal rats have microvessels < 20 μm﻿ in diameter and large blood vessels > 50 μm in diameter, with the former accounting for the majority of the total cerebrovascular system^34^. A previous study on the mouse brain used LSFM to analyze the vasculature in the entire ischemic brain^15^. In the ischemia mouse model, an overall loss of vascular density was observed and the average diameter of the microvessels damaged by cerebral ischemia was 10 μm. In addition, in the entire mouse brain, vessels with a diameter of 10–20 μm were less affected, whereas vessels larger than 20 μm in diameter were not significantly affected. Although the degree of infarction varies depending on factors such as species and degree of ischemia, our findings in the rat model are consistent with data from previous studies in mouse models. Therefore, microvessels that are 15 μm in diameter are significantly more damaged than larger blood vessels as a result of a stroke. Furthermore, the reduced rates of blood vessel loss were similar in the cortex and striatum, indicating that there were no region-related differences following stroke.

In this study, 3D reconstructions of thick tissues allowed us to measure vascular parameters while also measuring neuronal changes using immunostaining. Notably, both manual and automated cell counting with a program were used as a method for counting cells for quantitation. Cell counting was compared using random optical slices versus automatic cell counting using the program on tissue with a thickness of 500 μm. Except for a few errors, the quantitative results were nearly identical. We also found that severe stroke reduced the total number of vessels by 60%–70% when compared to the contralateral region, while it decreased the number and fluorescence intensity of neuronal-positive cells including NeuN, PV, and MAP2. However, we found that the fluorescence intensity of GFAP was higher in the ipsilateral region compared to the contralateral non-ischemic region.

Several studies have found that cerebral ischemia has a significant impact on neural and glial cells. It has been reported that the staining signal of NeuN-positive cells is weak but still present 24 h after a focal stroke, resulting in pyknosis^5,35^. We also found that only about 80% of NeuN-positive cells appeared in the contralateral region, and that morphological changes occurred. However, in severe MCAO, the number of NeuN-positive cells in the ipsilateral striatum decreases to 90% in the contralateral striatum on day 3^36^, implying that significant changes will occur after 24 h. PV plays a crucial role in the calcium homeostasis of the central nervous system^37^. Previous studies have reported that the number of PV-positive cells significantly reduces the damage caused by MCAO^38^. It is well understood that a decrease in the level of breakout during MCAO injury raises intracellular calcium concentration, resulting in neuronal cell death. This study found that 24 h after severe MCAO, focal ischemia causes a significant decrease in PV-positive cells in the cerebral cortex and striatum. Despite the lack of earlier temporal evidence in this study, PV-positive cells appeared to be more susceptible to ischemia within 24 h when compared with NeuN-positive cells. In the mature rat brain, MAP2 is a neuron-specific cytoskeletal marker that is primarily found in dendrites^39^. It is an early and exceptionally sensitive marker of ischemic neuronal injury after focal stroke^40,41^. Several studies have reported lower immunoreactivity levels for MAP2 in the infarcted area^41,42^. We found that the intensity of MAP2-related immunofluorescence in tissues 24 h after stroke decreased when compared to the contralateral region. We also discovered that 24 h after a severe stroke, vascular density was maintained at 60%–70% compared with the contralateral area. Based on the rate of decrease in vascular density, MAP2 and PV-positive cells were considered sensitive, while NeuN-positive cells were relatively resistant to ischemia. Glial cells, such as GFAP, are significantly affected by cerebral ischemia. An ischemic stroke, for example, is characterized by abnormal astrocyte activity, such as hyperplasia and hypertrophy, which increases GFAP immunoreactivity during the recovery process^43–45^. Although the intensity of GFAP-positive fluorescence increased in this study, the morphology of reactive astrocytes in the cortex could not be confirmed. As a result, it is assumed that severe astrogliosis forms a glial scar^46^. Furthermore, the clearing rate in the infarction region is assumed to differ from that in the contralateral region because of the glial scar.

Although the MCAO-induced vascular density and degree of nerve damage were dependent on the duration of occlusion^47^, it was demonstrated that total vessel length decreased compared with the contralateral region without a region-related difference; this was due to a similar decrease in the cortex and striatum. However, the number of PV-positive cells and GFAP-positive fluorescence intensity are more affected in the ischemic cortex than in the ischemic striatum. Conversely, NeuN-positive cells and MAP2-positive fluorescence intensity were not significantly affected. The striatum remains ischemic in MCAO with reperfusion, but the cortex returns blood to regulate its flow^48^. Thus, it is known that the ischemic core is striatal infarction, and cortical infarction causes delayed progressive neuronal cell death in MCAO^7^. However, no direct relationship was found between progressive neural death-related factors and MCAO-evoked neuronal markers across brain regions in this study. Neuronal markers such as PV and GFAP may be more affected by the physiological environment of the MCAO-induced rat cortical region.

In conclusion, we performed visualization and quantification of vascular and neural changes caused by MCAO in large volume tissues of a rat model. By combining 3D images with tissue clearing and automatic analysis programs, quantitative and statistically meaningful data for cerebrovascular diseases may be readily obtained.

## Materials and methods

### Animals

Adult Sprague Dawley rats (male, weighing 280–300 g; n = 10) were used for MCAO surgery. All studies were approved by the Animal Care and Use Committee at Kyungpook National University (permission No. 2020-0096) and conducted following National Institutes of Health guidelines for the use and care of animals and in compliance with the ARRIVE guidelines.

### MCAO surgery

Using a facemask, rats were anesthetized with 5% isoflurane in 2.4 l/min of oxygen. First, the left neck area with the common carotid artery (CCA) was incised vertically, and the lower surface fascia was also cut. Next, the left CCA, external carotid artery (ECA), and internal carotid artery (ICA) were exposed from surrounding tissues and dissected without damaging the adjacent nerves. To prevent bleeding, the lower part of the CCA (approximately 5 mm proximal to the carotid bifurcation) was clipped. To prevent blood backflow, a silk suture was tied around the ECA (approximately 5 mm distal of the carotid bifurcation). Next, a needle was used to make a small hole in the ECA (approximately 3 mm distal of the carotid bifurcation), and a filament (0.19 mm silicon-tipped monofilament, Doccol Corp., Sharon, MA, USA) was inserted through the small hole and introduced into the ICA lumen until resistance was felt. The filament was removed after 45 min of occlusion to allow for reperfusion. Following surgery, the wounds were sealed, anesthesia was ended, and the animals were placed on a warming pad for about 1 h to recover.

### TTC staining

The 2,3,5-triphenyltetrazolium chloride (TTC) staining is primarily used to visualize hypoxic tissue in the brain. The rats were allowed to live for 24 h before being sacrificed. Using the induction chamber, the rats were deeply anesthetized with 5% isoflurane in 2.4 l/min of oxygen. The rats were perfused with 1× phosphate-buffered saline buffer (1× PBS, pH 7.4) through the left ventricle-aorta, and the brains were then removed from the skulls. One rat was used as the TTC result as validation data, and the staining revealed ipsilateral brain infarction. A rat brain slicer (Zivic Instruments, Pittsburgh, PA, USA) was used to cut 2-mm-thick coronal slices to confirm the infarction site caused by MCAO. The sliced brain tissue was stained with 2% TTC (TTC; Sigma-Aldrich Inc., St. Louis, MO, USA) solution at 37 °C for 5–10 min.

### Lectin injection and perfusion

The following experiment was conducted to visualize and quantify the vascular and neuronal changes induced by MCAO. After anesthesia in rats, *Lycopersicon esculentum* agglutinin (tomato lectin, 50–100 μg/100 μl; Vector Laboratories, Burlingame, CA, USA) was injected directly into the left ventricle of the heart to stain the functional blood vessels^26^. After 2 min, the rats were perfused through the left ventricle-aorta. Each rat was fixed with 4% PFA after a pre-rinse with 1× PBS, and their heads were removed and placed in 4% PFA for 2–3 h. The brains were then removed from the skulls, post-fixed in 4% PFA overnight, and stored in 1× PBS overnight (pH 7.4).

### Tissue clearing

Using a rat brain slicer, we obtained 3-mm-thick coronal slices to visualize and quantify the vascular change. First, the brain slices were made transparent using a tissue clearing kit (Cat. HRTC-001, Binaree, Daegu, Korea). Briefly, the PFA-fixed brains were immersed in Binaree fixing solution for 24 h and incubated with tissue clearing solution in a shaking incubator at 37 °C for 4–5 days, and rinsed in a washing solution. Finally, the brains were incubated in mounting and storage solution (Cat. SHMS-060, Binaree) for 24 h for further clearing.

### Immunostaining

To identify neuronal damage after MCAO, we performed immunostaining with antibodies raised against neuronal and astrocyte markers. First, PFA-fixed samples were washed with 1× PBS and then sectioned into 0.5-mm-thick tissues using a vibratome (Leica, Vibratome VT1200, Wetzlar, Germany). Next, 0.5-mm-thick tissues were stained using Binaree Tissue Clearing for immunostaining (HRTI-001, Binaree) for easy antibody penetration into the thick tissue. Briefly, PFA-fixed brains were immersed in fixing solution at 4 °C overnight and incubated with tissue clearing solution A and B in a shaking incubator at 37 °C for 24 h. Primary antibodies and concentrations were rabbit anti-NeuN antibody (1:100, Abcam, Cambridge, UK), mouse anti-PV antibody (1:100, Swant, Bellinzona, Switzerland), Alexa Fluor 488 rabbit anti-MAP2 antibody (1:100, Abcam), and rabbit anti-GFAP antibody (GFAP, 1:100, Dako Agilent Technologies, Inc., Santa Clara, CA, USA). The secondary antibodies used for immunofluorescence detection were as follows: Alexa Fluor 488 AffiniPure F(ab′)_2_ Fragment Goat Anti-Rabbit IgG (H + L) (1:200; Jackson ImmunoResearch Laboratories, Inc., Bar Harbor, ME, USA) and Alexa Fluor 488 AffiniPure F(ab′)_2_ Fragment Goat Anti-Mouse IgG (H + L) (1:200; Jackson ImmunoResearch Laboratories, Inc.). The sliced brains were then incubated in mounting and storage solution (Binaree) for 24 h to match the RI.

### Image acquisition and 3D quantification

Images of 3-mm-thick brain slices (n = 3 animals) were acquired at a magnification of 5× using a Lightsheet Z.1 fluorescence microscope (Zeiss Corporation, Jena, Germany). Images of 0.5-mm-thick slices were acquired at 4× magnification using a Nikon confocal microscope (Nikon A1R confocal system, Nikon Corporation, Tokyo, Japan). The images for quantification and analysis were acquired using a 10× lens. Image rendering in 3D was performed using the Imaris program (version 9.5.1, Oxford Instruments, Abingdon-on-Thames, UK, https://imaris.oxinst.com). All parameters were measured in multiple ROIs (3–4 ROIs/each) in the ipsilateral cortex and striatum, where the vascular fluorescence intensity decreased rapidly near the infarcted border compared with the symmetrical lateral cortex and striatum. Quantitative analysis of blood vessels was performed as previously described^49^. Quantitative evaluation of vessel length and diameter was performed in *maximum intensity* projection mode with rendering quality set to 100%. The background subtraction option was selected to smoothen the image by creating a Gaussian filtered channel minus the intensity of the original channel (diameter = 2.02 μm). The filament module (no loop) was designed from a Gaussian filtered channel using a threshold algorithm to quantify vessel length and diameter (Supplement Fig. 1).

NeuN and PV-positive cells (n = 3 animals, respectively) in the ipsilateral and contralateral regions were quantified using National Institute of Health ImageJ software equipped with a cell counter plugin tool (version 1.53a, NIH, Bethesda, MD, USA, https://imagej.nih.gov/ij/) and the Imaris cell module. Furthermore, the MAP2 and GFAP-positive fluorescence intensities (n = 3 animals, respectively) in the ipsilateral and contralateral regions were measured using the NIS-Elements Microscope Imaging Software (version 5.2.1, https://www.nikon.com). Because the ischemic lesion distribution after MCAO was slightly variable, and cell counts and fluorescence intensity varied by region, we present the results as a percentage of contralesional regions. The total number of immunoreactive cells found in the contralateral region was expressed as 100%, and the number of cells found in the ipsilateral region was expressed as a percentage. The fluorescence intensities were compared to the contralateral area value, which was then normalized to 100%.

### Measurement of light transmittance

Transmittance curves for 3-mm-thick brain sections were obtained using a commercially available spectrophotometer (Lambda 950, PerkinElmer, USA). To obtain the collimated transmittance spectra (400–800 nm), adult rat brain sections were placed in a custom-made cuvette with a 5-mm optical path. Measurements were taken both during (after 24 h of clearing solution) and after clearing (matching RI) and divided into contralateral and ipsilateral sections.

### Statistical analysis

Statistical analysis was performed using Graphpad Prism (version 9.0, GraphPad Software Inc., San Diego, CA, USA, https://www.graphpad.com). The data of light transmittance are presented as means ± SD. The rest of the data are presented as means ± SEM. Two-way analysis of variance (ANOVA) and paired t-tests corrected for multiple comparisons by false discovery rate (FDR) were used for statistical evaluations. A p-value of ≤ 0.05 was considered statistically significant.

### Ethical approval

All applicable international, national, and/or institutional guidelines for the care and use of animals were followed. All procedures involving animals were done in accordance with the ethical standards of our institution and were approved by the animal rights committee at Kyungpook National University, Deagu, South Korea (permission No. 2020-0096).

## Supplementary Information

Supplementary Figures.

Supplementary Video 1.
