# Supplementary material for: Three-dimensional visualization of cerebral blood vessels and neural changes in thick ischemic rat brain slices using tissue clearing
Source: Sci Rep. 2022 Sep 23;12:15897. doi: 10.1038/s41598-022-19575-w (PMC9508267; doi:10.1038/s41598-022-19575-w)
Supplement: Supplementary file 1 — Supplementary Figures. [file 41598_2022_19575_MOESM1_ESM.pdf]

Supplementary information

Supplement Figure 1.

Quantitative analysis of blood vessels in MCAO-evoked rat brain slices using Imaris program. (a,d) Maximum intensity projection (z-stack) image of rat brain stack with microvasculature stained with tomato lectin (ROI = 1 mm × 1 mm × 1 mm). (b, e) Image stack applies the Gaussian filter to reduce the background intensity subtracted (diameter = 2.02 μm). (c, f) This image was acquired by the microvasculature filament tracer module of the microvasculature from the Gaussian filtered channel. Scale bar = 200 um.

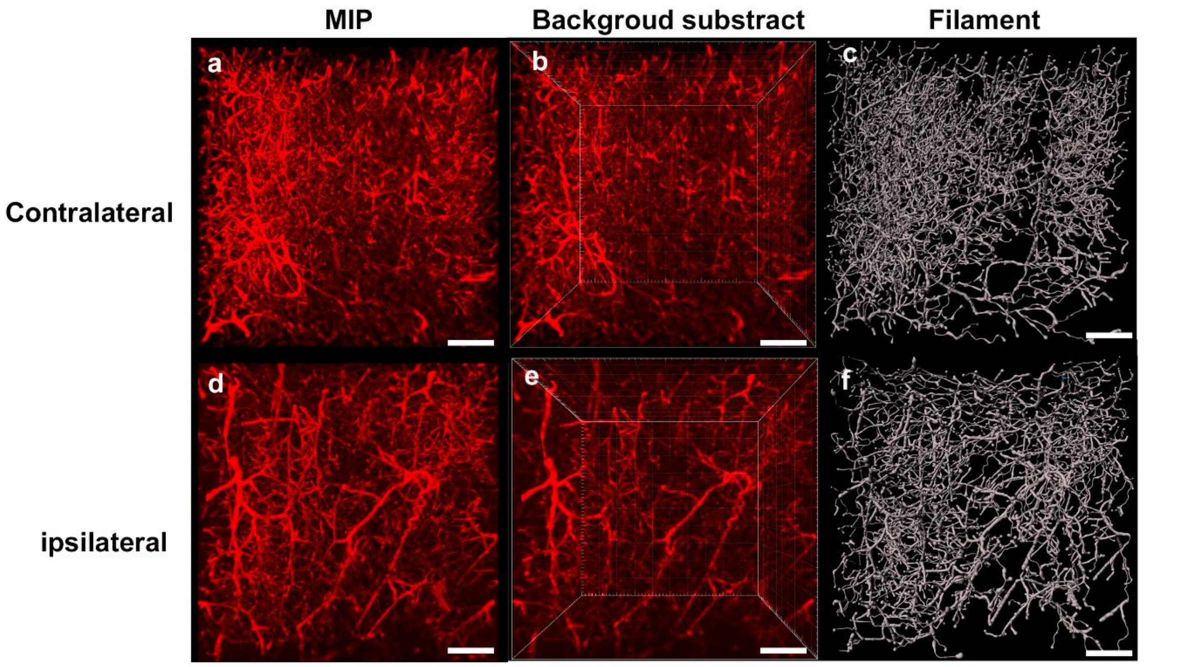

Supplement Figure 2.

Comparison of 2D manual counting and 3D automatic counting of neural cells at specific ROIs. (a, g) The 3D image indicates the ROI used to quantify NeuN- or PV-positive cells after 24 h of MCAO in rats. (b) A representative magnified view of a 3D block of NeuN-positive cells in the marked cortex 1 in several boxed areas in (a) is shown. (c) The five optical sections manually counted are indicated by a line. (d) The automated quantification analysis of the NeuN-positive cell in 3D was performed using a spot detection strategy generated using the Imaris v.9.5.0 software. (e, h) Graph of the ratio of NeuN- or PV-positive cells using manual cell counting in the ipsilateral areas to the corresponding contralateral areas of the cortex and striatum 24 h after ischemia (*n* = 5). Manual counting of NeuN-positive cells was performed by calculating the ratio of NeuN-positive cells in the ipsilateral region/contralateral region. No significant differences were observed in the ratio of NeuN-positive cells between the ipsilateral cortex and the striatum. Significant differences were observed in the ratio of PV-positive cells between the ipsilateral cortex and striatum. (f, i) The table shows the comparison of the ratio of NeuN or PV-positive cells in the ipsilateral areas to the corresponding contralateral areas of the cortex and striatum after ischemia by manual counting and automated counting. The manually counted data represent the mean ± SD. The automated analysis of NeuN-positive cells was performed using the Imaris software. * ROI = 0.9 mm × 0.9 mm, ** ROI = 0.9 mm × 0.9 mm × 0.5 mm. Scale bars = 1 mm in (a, g), 100 μm in (b-d). Paired t-test, FDR multiple comparison test, $^{###}$ $p < 0.001$, n.s., no significant difference.

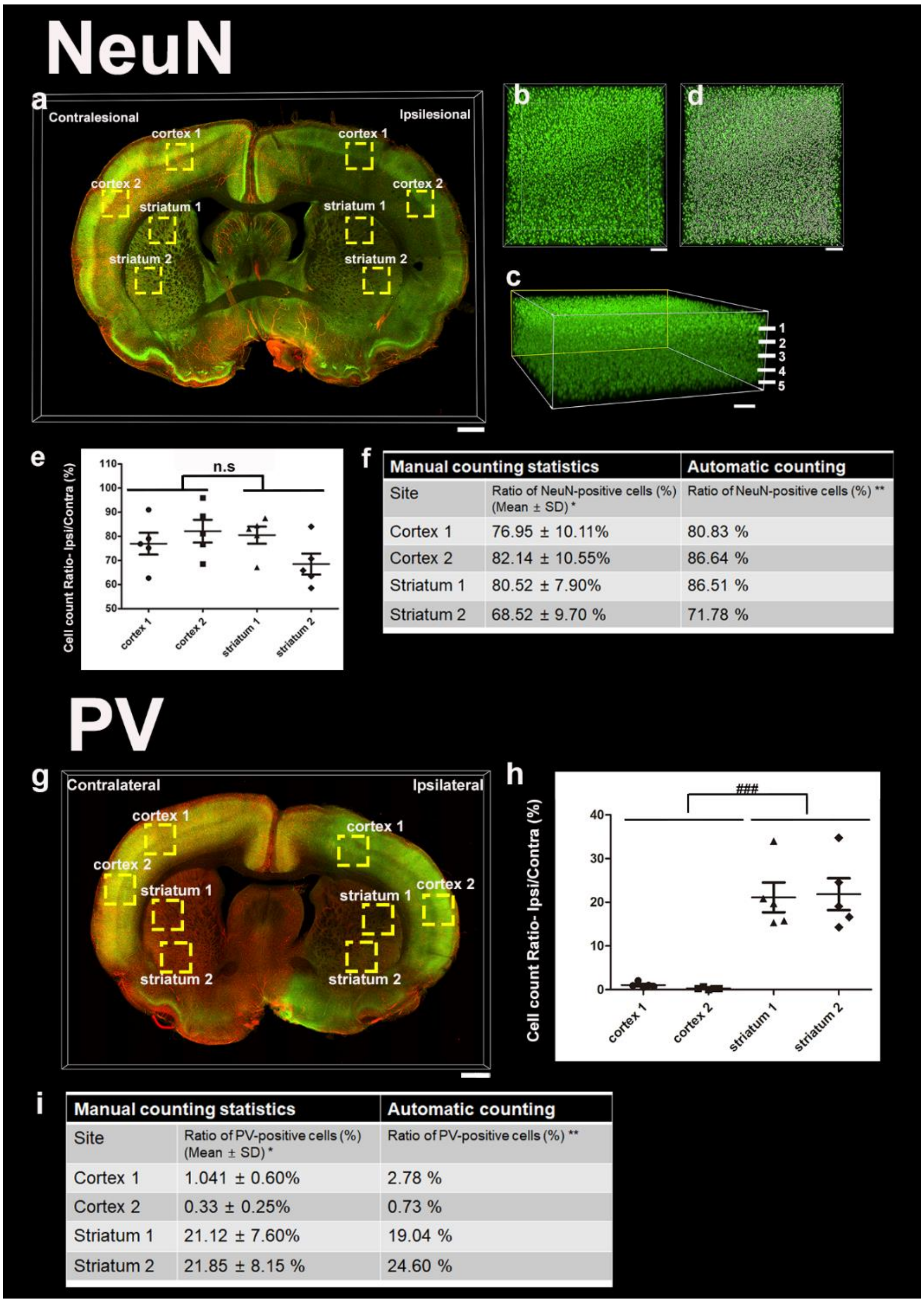

Manual counting statistics		Automatic counting
Site	Ratio of NeuN-positive cells (%) (Mean ± SD) *	Ratio of NeuN-positive cells (%) **
Cortex 1	76.95 ± 10.11%	80.83 %
Cortex 2	82.14 ± 10.55%	86.64 %
Striatum 1	80.52 ± 7.90%	86.51 %
Striatum 2	68.52 ± 9.70 %	71.78 %

Manual counting statistics		Automatic counting
Site	Ratio of PV-positive cells (%) (Mean ± SD) *	Ratio of PV-positive cells (%) **
Cortex 1	1.041 ± 0.60%	2.78 %
Cortex 2	0.33 ± 0.25%	0.73 %
Striatum 1	21.12 ± 7.60%	19.04 %
Striatum 2	21.85 ± 8.15 %	24.60 %

Supplementary Video 1. 3D visualization of the vasculature in the 3-mm thick MCAO rat brain slice stained with lectin.
